# Burnout and Brownout in Intensive Care Physicians in the Era of COVID-19: A Qualitative Study

**DOI:** 10.3390/ijerph20116029

**Published:** 2023-06-01

**Authors:** Léa Baillat, Emilie Vayre, Marie Préau, Claude Guérin

**Affiliations:** 1 Unité INSERM U1296—Radiations: Défense, Santé, Environnement, Université Lumière-Lyon 2, 69500 Bron, France; 2 CHU de Lyon, Groupement Hospitalier Centre, Hospices Civils de Lyon, 69002 Lyon, France

**Keywords:** burnout, brownout, intensive care physicians (ICPs), COVID-19, intensive care, qualitative research

## Abstract

The health crisis has had a strong impact on intensive care units. The objective of this study was to investigate the experience of resuscitation physicians during the COVID-19 health crisis to understand the associated determinants of quality of life, burnout, and brownout. This qualitative, longitudinal study covered two periods (T1, February 2021, and T2, May 2021). The data were collected in individual semi-directed interviews with 17 intensive care physicians (ICPs) (T1). Nine of the latter also participated in a second interview (T2). The data were examined using grounded theory analysis. We identified a multiplication of burnout and brownout indicators and factors already known in intensive care. In addition, burnout and brownout indicators and factors specific to the COVID-19 crisis were added. The evolution of professional practices has disrupted the professional identity, the meaning of work, and the boundaries between private and professional life, leading to a brownout and blur-out syndrome. The added value of our study lies in identifying the positive effects of the crisis in the professional domain. Our study revealed indicators and factors of burnout and brownout associated with the crisis among ICPs. Finally, it highlights the beneficial effects of the COVID-19 crisis on work.

## 1. Introduction

Good health in the workplace is defined as a state of well-being; it is dependent on the repercussions of working conditions on health [1]. Over the last several years, many professions have been increasingly affected by psychological health problems at work [2]. The ongoing COVID-19 pandemic has led to even greater concern about well-being in the workplace.

In countries around the world, the different waves of COVID-19 infection have meant that the pandemic curve and the proportions of the population affected have varied over time [3,4]. In France, epidemiological data at the start of the third wave (first data collection (February 2021)) revealed an increase in COVID-19 cases and a high mortality rate. The Ministry of Health was forced to adapt emergency health measures to fight the pandemic [5], and it demanded that hospitals, in particular intensive care units (ICUs), deploy considerable resources. The consequence was that ICU personnel—and more in particular, the intensive care units—had to deal with a fracturing health system [6]. Resuscitation is a specialty that was previously unknown to the general public [7]. However, the ICU staff has been at the forefront of the management of the most-severe cases of COVID-19. In France, between March and June 2021, nearly 106,000 patients with COVID-19 were hospitalised in intensive care units, slightly more than half of them in resuscitation service [8]. In addition, in the third wave, transfers of patients to less-affected regions were more difficult to achieve, putting pressure on bed occupancy [9].

The global health care situation led some intensive care staff to take time off from hospital work or to resign [10], resulting in a shortage of human resources. The major reorganisation of care tools, the fear of being contaminated, the massive influx of patients, the challenge of the care system, and the confrontation with the stress of the general population are among the main factors of psychological suffering observed among ICPs [11]. Continuous waves of infection have led to increased exhaustion and burnout for ICPs [12]. Faced with continuous and repeated stress, they also expressed a feeling of weariness in their jobs, leading them to question the meaning of their work [13].

It was in this relatively tense context that the WHO issued an alert on the dangers of burnout among doctors and care providers [14]. 

The concept of burnout was introduced in the 1970s by Freundeberger [15]. Burnout is characterised by a state of professional exhaustion resulting from chronic and prolonged exposure to stress and “emotionally” stressful work situations [16]. Burnout is defined by three aspects: emotional exhaustion (lack of energy, exhaustion of emotional resources, and demotivation), depersonalisation (development of impersonal, detached, and negative attitudes), and the lack of personal and professional fulfilment (devaluation of one’s work and skills) [17,18]. In France, the Direction générale du travail in collaboration with the Institut national de recherche et de sécurité (INRS), the National Agency for the improvement of Working Conditions (Anact), and a college of experts (researchers and practitioners) have inventoried the individual and collective indicators of this syndrome [19] (see Table 1).

The main factors of burnout are work overload, lack of control, insufficient compensation, collective collapse, procedural and organisational justice violations, and value conflicts [20]. In the context of ICUs, specific professional burnout factors have been described [21,22] and categorised into four major dimensions [23] (see Table 2).

According to a study conducted in 85 countries, between April and May 2020 (i.e., the first wave), 50% and 30% of ICU staff showed symptoms of anxiety and depression, respectively [24]. In the context of COVID-19, the study suggested that six determinants were at the origin of these symptoms as follows: the fear of being infected, the inability to rest, the inability to take care of one’s family, struggling with difficult emotions, regret over restrictions imposed on visits to the ICU, and witnessing rushed end-of-life medical decisions [24,25]. The confrontation with a little-known virus and the overexposure to patients’ deaths have psychologically affected health care professionals [26] and degraded the feeling of the usefulness of the health care function [27]. In addition, this exceptional context resulted in numerous moral dilemmas being raised in ICUs [28], which may have led personnel to question their professional identity. Consequently, it was possible their previous representations of their profession came into conflict with the realities of their work, increasing the risk of burnout [29].

Other risks weighed particularly heavily on the meaning of work during the study period. Brownout, which is a type of professional exhaustion caused by a loss of sense of work, is one example. Brownout was theorised in 2016 by Mats Alvesson and Andre Spicer as a devastating long-term syndrome for the organisation and for the psychological health of individuals [30]. It is defined as “a professional ill-being due to the loss of sense in the face of the tasks to be accomplished” [31], p. 164. Table 3 brings together all the brownout indicators identified by several authors in the field [30,31,32,33,34] (see Table 3).

As for the brownout factors, they mainly refer to the absurdity and uselessness of the tasks assigned, the lack of understanding of the work to be performed, the lack of stimulation at work, the mismatch between work and personal values, and the ethical conflicts generated by work [30,31,32,33,34].

Burnout and brownout can be difficult to differentiate since they share common manifestations with similar determinants. Brownout is more difficult to discern because it is a factor of mental resignation and where the question of work values are central. Moreover, the more the change or the evolution in a situation is felt as radical, the greater is the risk of a loss of meaning and values [35].

Therefore, the major and abrupt transformations imposed by the onset of the health crisis and its prolonged nature increased the risk of brownout. The deterioration of working conditions gave rise to the emergence of inner conflicts, both the “desire to save and the impossibility of doing so” [36], p. 181, the perception of a loss of interest in work, and the impression of a situation with no end in sight [33]. In the present study on ICPs, the overcrowding of hospitals and the management of serious, clinically identical disorders tended to lead to a professional activity that was cyclical, continuous, and devoid of meaning.

In France, the third wave resulted in continued psychological, emotional, and physical suffering of ICU personnel. Before the COVID-19 era, the ICU was a sector already known for its high rate of professional burnout [37]. The existence of pre-existing stress factors in this population combined with the pandemic context suggests the importance of examining the health of these care providers in the era of COVID- 19, especially since professional burnout is a factor linked to the deterioration in the quality of care delivered to patients [38].

The objective of this study was to investigate the experience of ICPs during the COVID-19 pandemic in order to understand the mechanisms involved in the deterioration of their mental health and to identify the determinants of the occurrence of burnout and brownout. Specifically, we studied two types of ICP in France: intensive care anaesthesiologists and intensivists. Anaesthesiologists work in the operating room, in surgical ICUs, and in the field of pain management. They also provide post-operative and post-trauma care to patients. Intensivists are involved in the diagnosis and management of all vital function failures of medical origin and work in medical ICUs [39].

## 2. Materials and Methods 

### 2.1. Study Environment

In the Grand Est region of France, these COVID-19′ admissions represented 60% of hospital capacity, and a state of emergency was announced for the region’s health system [40]. In March 2020, the French hospital system was plunged into a critical situation. However, the implementation of the first lockdown on 17 March 2020 helped to reverse the trend in hospital admissions [41]. However, this improving trend was not seen in the health establishments of France’s Grand Est region (geographic zone of our study). There, the peak of the third wave saw an infection rate of 380 cases per 100,000 inhabitants. When, in May 2021, these figures decreased, with approximately 150 cases per 100,000 inhabitants [42], the rate of intensive care hospitalisations in the region increased. More specifically, the number of COVID-19 patients hospitalised in intensive care units almost doubled between February and May 2021 [42,43]. The impact of the third wave on hospitals in the Grand Est region was huge, due to rapid congestion from the massive influx of patients and the reorganisation of the workloads and working times for intensive care physicians (ICPs).

### 2.2. Study Design and Setting

We conducted a longitudinal qualitative study that used “comprehensive logic” [44], p. 25. Because of the spatiality and temporality of the COVID-19 pandemic, a longitudinal approach is the most0adequate choice to provide a “temporal dimension of social facts” [45], p. 312. The comprehensive logic approach made it possible to come as close as possible to human experiences and motives, in their singularity [46]. We focused on the subjectivity of ICPs’ experience of the pandemic, as professionals and as persons, by focusing on the meaning they attributed to this experience. In addition, as the health crisis constituted a complex, uncertain, and little-understood context, an exploratory qualitative investigation was a particularly relevant choice for producing knowledge on unknown phenomena [47]. 

More specifically, the study aimed to identify, through the experience of caregivers working in an unprecedented context of a pandemic and which was being established over time, the appearance of and factors associated with burnout and brownout. As major differences in the pandemic curve were observed in the different regions of France, we analysed the data in terms of a specific geographic location (i.e., the Grand Est region) to ensure the homogeneity of the sample, with a view to improve the internal validity of the results [48].

The first part of the study was conducted at the beginning of the third pandemic wave (T1, February 2021) at a moment when the impact of the epidemic on the health system in the Grand Est region was considered significant and the territory was placed in a state of emergency. The second part of the study was conducted at the end of the third wave (T2, May 2021), when vaccination had just commenced for all persons living in France over the age of 18, providing the prospect of an improvement in the crisis situation.

### 2.3. Participants

We interviewed qualified anaesthesiologists and intensivists and residents from several medical and surgical ICUs in public hospitals in a large metropolis in the Grand Est region. The study inclusion criteria were: (1) being a qualified medical anaesthesiologist, or intensivist, or intern; (2) participating (at least for a time) in the management of the COVID-19 health crisis; (3) practicing in one of the medical or surgical ICUs in the region.

### 2.4. Data Collection and Analysis

The study was based on semi-structured interviews. For T1 (February 2021), the interview guide aimed to understand: (1) current professional activity and workload, (2) the relationship to work/profession and any intentions to leave one’s position in order to work elsewhere, (3) emotional regulation linked to work activity, (4) the collective and relational dimension of work, (5) ethics and professional standards in the context of the pandemic, and (6) the relationship between working life and private life. For T2 (May 2021), these same dimensions were investigated in order to understand their potential evolution (see Table 4. All interviews were recorded and fully transcribed. 

Once the corpus was constituted, we used grounded theorisation, which produces theorisation since it aims at questioning or completing theoretical elements [49]. According to this approach, each interview was processed in its entirety and units of meaning were identified by the researcher. These units were compared to each other in order to develop categories in a process of conceptualisation. This process, specific to grounded theory, is a deductive process at the service of an essentially inductive approach [50] based on the subjective experience of the participants. The researcher continually moves back and forth between inductive thinking (developing concepts, categories, and relationships from the text) and deductive thinking (testing concepts, categories, and relationships against the text). This work corresponds to an axial coding defined as a “*complex process of inductive and deductive re-thinking*” [51], p. 114. Thus, the conceptualisation of the data, which represents the deductive aspect of the approach [51], progresses as the inductive analysis of the interviews is conducted. The relationship of theory to empirical work can be summarised as follows “*The principle of openness implies, that the theoretical structuring of the issue under study is postponed until the structuring of the issue under study by the persons being studied has emerged*” [52], p. 343.

In summary, the analysis was carried out according to these two specific and intersecting coding principles: inductive coding (categories related to the data) in order to successfully extract meaning from the raw data and deductive coding (categories related to the concepts and theories) in order to describe and evaluate the processes and behaviours involved in the evocation of items applicable to each category. This double codification allowed for a broader view of the phenomenon studied.

At the end of the analysis of the constituent corpus (T1), more central dimensions than we had anticipated emerged from the analysis of the participants’ discourses. They regarded: (i) response-shift, implying that the changes observed did not reflect the real change felt, (ii) methods of psychosocial adjustment in the face of a situation that was unprecedented in terms of its duration, and (iii) unexpected contributions and benefits that the crisis brought about at the intra-individual, inter-individual, and professional levels. Consequently, the dimensions of the interview guide investigated in T1 (February 2021) were completed in T2 (May 2021) by these three new components (see Table 4). The analysis of the corpus of data for T1 and T2 was carried out according to the same method.

This study was authorised by the Ethics Evaluation Committee of Inserm (Decision No. 20-759, IRB00003888) and was declared to the Data Protection Officer of Lyon 2 University.

## 3. Results

Seventeen ICPs (12 qualified and 5 residents), with a median age of 35 years (σ = 9), including 12 men and 5 women, agreed to participate in T1 (see Table 5). Three months later, 9 ICPs agreed to participate in T2 (6 qualified and 3 residents; see Table 6). Of these, six were males; the median age was 32 years (σ = 4).

Using indicators from the literature and an initial examination of the corpus of data collected, we constructed an analysis grid composed of four categories. The following topics were discussed: (i) the socio-health context and organisation of work during the CO-VID-19 pandemic, (ii) the relationship with intensive care patients and their relatives, a new approach in the era of COVID-19, (iii) multiple disorders of mental health: between burnout and brownout, and (iv) resources contributing to maintaining good mental health. The construction of these categories took into account the six steps of grounded theory: codification (labelling all the elements of the initial corpus), categorisation (naming the important aspects of the phenomenon), linking, integration (grasping the meaning of the essence of the subject), modelling (reproducing the dynamics of the analysed phenomenon), and theorising (careful and exhaustive construction of the multidimensionality and multicausality of the studied phenomenon) [53]. Each of the categories was subdivided into subcategories (with the exception of Category 4; see Table 7). An analysis of their relationship with existing theories (theorising) was carried out. The categories and sub-categories were set against a theoretical reasoning based on a “recursive cognitive” process made by repeated back and forth between data and theories [54].

### 3.1. Socio-Health Context and Organisation of Work during the COVID-19 Pandemic 

Respondents described several factors and indicators influencing their health during the COVID-19 crisis. They detailed the repercussions on their well-being, both professionally and personally.

#### 3.1.1. Work Uncertainty and Adaptation to Change

T1 (N = 17): Thirteen ICP mentioned major and permanent reorganisation of their work that influenced the functioning of the health establishments where they worked and that had a great impact on professional practices. For nine of them, this reorganisation necessitated a strong capacity to adapt and great cognitive and physical efforts, especially as their supervisors and departments had heterogeneous expectations of them. ICU understaffing and high turnover led to the creation of inexperienced teams who found it difficult to manage the pandemic. Eight ICPs mentioned that the lack of human and material resources affected the quality of care and work. Finally, five residents talked about the impact of the pandemic on their training and their concern for their professional future.

T2 (N = 9): In T2, the difficulties associated with the major organisational changes and the lack of resources were less frequently mentioned. The impact on training was an important element in residents’ discourses. In addition, several participants mentioned a loss of meaning to their work because of organisational changes, which often did not reflect standard professional ICU working norms.

*It’s true that because we are still training to become doctors, well, I’m a bit worried that I’ll be less well trained in these [previously mentioned] problems than in COVID problems.*

Male, 25–35 years old (T1)

#### 3.1.2. Perceived Characteristics of the Work Environment

T1 (N = 17): Fifteen ICPs mentioned increased hours and workload, as well as responsibilities, which led to role conflict. For four residents, work demands had become exacerbated and superiors more intransigent. The arrival of support staff with no ICU training and the overbearing and uninterrupted management of patients with a new pathological profile intensified for six ICPs’ mental load and workload. The use of information and communication technologies (ICTs) to provide support and information to patients’ families meant that a greater amount of time was needed to keep them updated on their hospitalised relative’s health situation. Nine ICPs empathised with families suffering from not being able to visit their loved ones. Finally, the perpetual contact with bereavement constituted a heavy emotional burden.

T2 (N = 9): The increase in work demands was a prominent sub-category in the discourses. Patient care was problematic and the management of patients’ families increasingly difficult, because of a globally negative change in their behaviour. Nevertheless, constraints related to ICPs’ workload and to the mental load arising from the rupture of the family–patient link (i.e., families not being able to visit their hospitalised relatives) were less strong than in T1.

*Well, these are difficult times, because we have a greater workload (…) the patients are all the more demanding (…) sometimes [these difficult times] a little complicated on a human level.*

Male, 25–35 years old (T1)

*But there were a lot of really aggressive families (…) it was really claim (…).*

Female, 25–35 years old (T2)

#### 3.1.3. Social Climate and Well-Being at Work

T1 (N = 17): For 13 participants, the work social climate deteriorated because of changes in the behaviours of care providers, who became impersonal and negative, with breakdowns at the communication and relational levels. The break-up of collective working values hindered proper care management and consistency in decision-making. Four ICPs mentioned that health protocols and social restrictions weakened relationships with patients and their families, as well as relationships between colleagues, as they prohibited moments of sharing. Finally, the lack of appreciation by the management of the work performed by care providers, especially with regard to residents, reinforced the negative atmosphere.

T2 (N = 9): Negative behaviours continued, with a phenomenon of depersonalisation. For two ICPs, the crisis caused both a decrease and a surplus of communication and of transmission of information, each of which gave rise to misunderstanding and tension in the teams. Added to the lack of consideration for others was a lack of recognition for four ICPs; this sentiment was all the stronger since the ICPs felt that the gratitude of the French population as a whole had dissipated. This lack of recognition, associated with a negative social climate at work, led the ICPs to question their usefulness and their roles in managing the COVID-19 health crisis.

*However, it still exacerbated uh… also tensions and personality types from what I was told (…) people who were quite blocking, who never made things easier.*

Female, 35–45 years old (T1)

#### 3.1.4. Work–Life Balance

T1 (N = 17): Thirteen ICPs considered that health restrictions on private life deprived them of daily pleasures and were an obstacle to long-term self-projection. The pastimes that usually allowed them to disconnect from work and enjoy social interaction were taken away from them. For nine ICPs, time constraints upset their work–life balance, and four participants saw their sleep disturbed by thoughts about difficult professional situations. Finally, since the start of the pandemic, five ICPs had been cautious about interacting with their relatives for fear of contaminating them; sometimes, they voluntarily shut themselves away, which led to a feeling of isolation.

T2 (N = 9): Despite better knowledge about the disease and vaccination, the problem of disconnection persisted for four ICPs, as did the cautious behaviour toward interaction with relatives. The upheavals to private life were discussed differently, as if they were events from the past; this was most likely due to the easing of health and restrictive measures. Nevertheless, the workload still disturbed the work–life balance for five participants, to the point where the distinction between both became blurred.

*It was the time; we spent so much time in the hospital that the little we spent at home was for essential things, for eating, sleeping, taking a shower (…) and we started [all over] again.*

Female, 25–35 years old (T2)

### 3.2. The Relationship with Patients in ICU and Their Relatives, a New Approach in the COVID-19 Crisis Era

In addition to material and organisational upheavals in working conditions, the ICPs interviewed emphasised relational aspects. The accelerated modernisation of digital technologies transformed the relationship and communication with families. Furthermore, the sedation of COVID-19 patients made communication even more difficult.

#### 3.2.1. The Use of Information and Communication Technologies with Patients’ Families

T1 (N = 17): For ten ICPs, the difficulty was providing satisfactory information about the monitoring of a patient’s condition to their family and friends because they (i.e., the ICPs) had to use information and communication technologies (ICTs) as a new means of care support, given the restrictions on hospital visits. The latter regulation, at odds with what is considered “ethically acceptable” always from the point of view of ten participants, led to ethical conflicts and self-questioning about the human dimension of their profession. Eleven ICPs considered ICT a major constraint, because, among other things, of the lack of non-verbal language and the lack of adaptation of speech, which weakened their relationships with patients’ families and led to an increase in conflict.

T2 (N = 9): A decrease in ethical conflicts and ICT-related difficulties was observed in six participants, possibly because of the partial reopening of the doors of the hospital to family visits and to the familiarisation of doctors with ICT. For five ICPs, the incomprehension of families regarding restrictions led to their (i.e., the families) becoming increasingly aggressive and invasive.

*It [ICT] doesn’t go down well, and it harms families’ understanding of the situation. Uh, especially when we come to the moment when, uh, we discuss treatment interruptions, uh… well the fact that the families haven’t seen the patient, haven’t see him deteriorate (…) it doesn’t help us work.*

Male, 35–45 years old (T1)

#### 3.2.2. Quality of the Care Provider–Patient Relationship

T1 (N = 17): The fact that the majority of COVID-19 patients in ICU care were placed in an artificial coma (intubated) and/or in very serious condition weakened the doctor–patient relationship. Consequently, eight ICPs effectively developed a care-only-based relationship with patients, as opposed to one that also included a personal dimension. Even when they were awake, the high turnover of patients meant there were few opportunities to establish any type of relationship beyond simply providing care. For six ICPs, these dimensions were obstacles to efficient communication in the context of therapeutic follow-up, their fear being that they would forget that each patient has a unique personality.

T2 (N = 9): Relational difficulties related to unconscious patients were less present. However, they could still exist when patients were awake, as their capacity for expression was weak due to the aggressiveness of the care provided. To tackle this, some doctors decided to spend a considerable amount of time beside the patient’s bed; some did this to support patients who felt lonely because of the absence of their families.

*Well, you get the feeling you’re entering the room and patient number 1 is on a respirator, patient number 2 is on a respirator, etc. and you… it’s totally impersonal in the end.*

Female, 25–35 years old (T1)

#### 3.2.3. Ethical Issues of Care and Access to Care in the Pandemic Context

T1 (N = 17): The need to constantly make treatment decisions aroused complex emotions in the study’s ICPs, given the high patient mortality rate. Decisions were sometimes made at inopportune times and in an unfavourable environment. Two residents said they were not able to endorse decisions made by multidisciplinary teams. Tensions and fatigue could influence decisions, which could become irrational. Ethical conflict arose when doctors wanted to defend their ideologies and not participate in choices they considered illegitimate. Six ICPs mentioned patient selection (i.e., deciding who would and who would not be provided care) due to logistical constraints. Finally, four ICPs spoke of the brutal impact of the treatment administered to the patients. The line between therapeutic obstinacy and human preservation was blurred.

T2 (N = 9): The exacerbation linked to decision-making and ethical conflicts was a little more frequent in the discourses for T2. Five ICPs stated that therapeutic obstinacy was a reality that led care providers in general to question their practices. However, some were unsure as to whether they themselves had practiced therapeutic obstinacy. This finding is proof of the difficulty in defining ethical limits of care during the COVID-19 crisis. For two ICPs, unlike for T1, patient selection enabled the preservation of human dignity and the provision of intensive care to patients at risk.

*And, uh, some of my colleagues tend to give up on some patients (…) I have a lot of colleagues who, uh, want to stop after two weeks, uh, raise their eyebrows… when we [all] know that it’s a disease that takes time to heal.*

Male, 25–35 years old (T2)

### 3.3. Multiple Disorders of Mental Health: Between Burnout and Brownout

As a reminder, the burnout indicators were divided into three categories: related to the functioning of the organisation; related to the health and safety of workers; corresponding to individual signals [19]. In addition, four main burnout factors were identified in the scientific literature in this field: organisational, relational, care-related, and workplace-related factors [23]. Finally, concerning brownout, the main indicators corresponded to a decrease in motivation, a feeling of lassitude and being disillusioned and downhearted, dissatisfaction, and uncertainty about and the absence of perspective in one’s professional career [30,31,32,33,34]. The main factors were the uselessness and absurdity of the tasks to be performed, the performance of non-stimulating tasks, and the inadequacy of the work to personal values and ethical conflicts [30,31,32,33,34].

Our results confirmed the presence and relevance of the indicators and factors of burnout and brownout considered. The analyses carried out allowed us to operationalise and identify the occurrence of burnout and brownout, but also the characteristics of the risk situations and the specificity of the factors at work (e.g., ethical issues or work environment; see Table 8) according to the period considered (T1 and/or T2).

Our results revealed the presence of burnout indicators in 11 participating ICPs, expressed in particular in terms of physical and mental fatigue (T1 and T2), while burnout risk factors were detected in 16 participants (see Table 9). Work overload (T1) and emotional demands (T1 and T2) were the main determinants of burnout.

Compared to the workload, it’s not so much, uh, *respondent inhales* it’s not so much the, the stress, it’s more, uh, it was more, really, physical fatigue.

Male, 25–35 years old (T1)

*We had fairly close relationships with the people we had on the phone; since they were on the phone every day, they confided things to us; sometimes we happened to have families of people who were themselves sick so they…we called them.*

Male, 25–35 years old (T1)

**Table 9:** Presence of burnout and brownout indicators and factors for T1 in 17 participants.

Type of Exhaustion	Burnout		Brownout	
Risk indicators and factors	Indicators	Factors	Indicators	Factors
No. pers. concerned out of a total of 17 participants	11	16	11	13

Note. The number of participants who mentioned a burnout or brownout factor or indicator at least once was counted. For example, in the “burnout/indicators” column, 11 participants mentioned a state or feeling characteristic of the onset of burnout.

During the third wave of COVID-19, we identified brownout in 11 participants (see Table 9), the main indicators being a feeling of weariness and uselessness (T1 and T2) and demotivation (T2). We also identified factors likely to generate brownout in 13 participants (see Table 9), the most important of which were a loss of beliefs in values, in terms of tasks that conflicted with ethics (T2), repeated confrontation with death, and the repetition of work given the homogeneity of the patient profile (T1).

*Then, there’s a kind of weariness that sets in because you realize that you, your, your medical work is very, very repetitive, uh, it’s a little unsettling to take care of…systematically, patients with the same disease.*

Male, 25–35 years old (T1)

*Our beds are full all the time, [you feel] that the disease is still just as serious, that the patients, they just die; you get the impression you’re not of much use.*

Female, 25–35 years old (T1)

Although our sample was limited, it seems that the factors at the origin of the onset of exhaustion were likely to vary in nature and intensity according to the study period and the form of exhaustion (see Table 8 and Table 10).

No burnout factor prevailed at the end of the third wave (T2), and specifically, work overload—the principal determinant at the beginning of the third wave (T1)—was less obvious. With regard to brownout factors, the performance of tasks, decision-making, and compliance with rules that clashed with ethics were still present in May 2021 (T2), while confrontation with death, as a risk factor, was less present.

At the end of the third wave, we also observed a decrease in the presence of exhaustion indicators in participants’ statements, irrespective of the type of exhaustion (see Table 10). The reduction in the number of hospitalisations, the easing of the ban on family visits, and the reduction in mental load and workload during these visits most likely contributed to this attenuation. However, our results showed that brownout indicators were still as present as burnout indicators (see Table 10).

Well, in any case, recognition, we’ll never get it *laugh*, uh, it’s more like [financial] support saying, ‘take a little money and shut up’.

Female, 25–35 years old (T2)

*What is problematic is that there are no visits (…) we know that it’s almost inhuman, when all is said and done, not to open visits to families in contexts like that.*

Female, 25–35 years old (T2)

Our results demonstrated the value in understanding—in the context of future research—not only the two different forms of exhaustion and their relationship, but also their respective determinants and their variability over time.

### 3.4. Benefits of the Pandemic on a Professional Level

Ultimately, while our results demonstrated the risks of the COVID-19 pandemic in terms of good health, defined by the World Health Organization (WHO) (Preamble to the Constitution of the World Health Organization, as adopted by the International Health Conference, New York, 19–22 June 1946; signed on 22 July 1946 by the representatives of 61 States (Official Records of the World Health Organization, No. 2, p. 100) and entered into force on 7 April 1948) as “a state of complete physical, mental and social well-being and not merely the absence of disease or infirmity”, they also attested to the unusual benefits for ICPs. Following the analysis of the first data collection (T1), we identified additional results in the corpus that referred to the positive effects of work in the context of the COVID-19 pandemic, for the most part at the professional level.

Ten ICPs said that they developed knowledge and skills during the management of the health crisis, an event that they regarded as an exceptional experience. The COVID-19 pandemic offered them the possibility to acquire the basic rules of health crisis management, on a practical and theoretical level. The ICPs, a population driven by a thirst for learning, were satisfied with the professional know-how acquired, considering it as an enriching experience whose benefits could be used in the future.

*As a result, we had a lot of chronic ICU patients, and indeed, I believe, yes, that I acquired skills and, yes, medical skills for these chronic patients, a bit of intensive care.*

Male, 25–35 years old (T2)

Three ICPs felt that they had managed to adapt and to perform their work in a consistent fashion in a difficult professional environment. This reinforcement of a feeling of self-efficacy illustrates positive achievements and successes, through the mobilisation of necessary resources and a belief in their abilities. Recognition of their own acquired skills and abilities generated a degree of perseverance and played a crucial role in work engagement and performance.

*It reassured me about my ability to, uh, see that I could, uh, adapt to a situation, that is to say to change medical practice, because for us, it is a big change in practice.*

Male, 35–45 years old (T2)

Furthermore, the crisis lay at the origin of an awareness relating to several professional aspects: the need for teamwork, the importance of the quality of life at work, and the appreciation of the social bond with others. For six ICPs, the difficulty in implementing these aspects during the crisis generated a recognition of their importance; accordingly, these aspects became a real need for them. Instead, the emergence of poor work practices and new ways of working helped the ICPs become aware of professional practices to avoid and the limits of the ICU and of the ICP profession.

*The, the patients we take care of, they are… either they die, or they are… they remain very serious for a very long time; they remain very ill for a very long time (…) that, that made me realize a little, that what we do is not… it’s not magic.*

Male, 25–35 years old (T2)

For three participants, the crisis could create meaning (e.g., conducting scientific studies on COVID- 19, participation in the collective effort, satisfaction from having more duties). One ICP stated that the pandemic and the changes in work organisation enabled him to find harmony with his professional identity, which had been lost until then; this harmony was the result of COVID-19-related changes to the hospital system, which placed the human dimension and the fundamentals of medicine back at the centre of attention. Finally, for five ICPs, the lessons to be learned from the crisis constituted an element of hope and fundamental support for preparing for future crises—in terms of understanding and managing them—and for improving the work environment.

*It made it possible to be…to refocus on the priorities of our profession, that is to say that everyone went back to treating people, that’s still why we’re doctors (…) So we rediscovered our profession.*

Male, 35–45 years old (T1)

These various benefits brought about by the COVID-19 crisis are factors of well-being that offered, at least temporarily, different resources to improve the working environment. In this sense, we assumed that these perceived subjective benefits may have reduced burnout and brownout.

## 4. Discussion

Our comprehensive approach aimed to explore the lived experience of ICPs working in medical and surgical resuscitation in the context of the COVID-19 health crisis and to understand the effects on their mental health (burnout and brownout).

This study confirmed that the characteristics and evolution of the socio-sanitary context and working conditions in France brought about by the COVID-19 pandemic particularly affected the mental health of ICPs. Prior to the crisis, the ICU was already recognised as a sector where the prevalence of burnout in ICPs was high (between 25 and 50%) [55]. Factors related to the organisation of work, the quality of professional relations, the quality of care and the work environment, as well as collective and individual indicators had already been identified before the COVID-19 crisis in the field of intensive care and, more broadly, among health professionals [19,23]. Yet, during the pandemic, the risk and the severity of these determinants increased [11]. However, other factors and indicators specific to the crisis were identified in our study and support recent findings in the field of occupational psychology. Specifically, these factors include restrictions on ICU visits and hasty medical decision-making, but also multiple reorganisations and related uncertainty at work (new services, new clinical situations, lack of equipment, etc.) [24,56]. Our study highlighted that, at the organisational level, the consequences of changes in the organisation of work generated tension. 

In line with previous findings [57], our data showed that incompatibility between defined roles and assigned tasks can lead to instability and discomfort. Taking on new tasks or tasks to be performed in unusual conditions requires the acquisition of specific skills. It also increases the risk of role conflict, conceived as “the incompatibility between two or more expectations and/or demands, such that acquiescence to one of them, on the part of the intended incumbent, makes the acceptance or fulfillment of the other more difficult if not impossible” [58], p. 184, which in turn can lead to burnout [59]. Furthermore, in the discourse of the ICPs, we observe the characteristic effects of organisational change, an alternation between loss and regaining of meaning in work, with an increased risk of distress and deterioration in performance [60].

However, if we consider the nature of the burnout factors [23], we observed between T1 and T2 a disappearance of several factors related to the work environment (e.g., equipment problems) and work organisation (work schedules, admissions, requisitioning of inexperienced staff, etc.). As for the indicators selected, our results showed a dissipation in T2 of the risks linked to the functioning of the structure identified in T1 (e.g., staff movements). This trend, observed in four of our participants, can be explained by the decrease in the number of people newly hospitalised in the intensive care unit observed from April 18, 2021 [42]. Indeed, in May, 389 people were hospitalised in intensive care, while the initial capacity of the Grand Est region before the crisis was 471 beds [61]. It can also be assumed that, over time, health care personnel have developed hospital resilience and have adapted to change [62].

At the relational level, it appears that confrontation with an unusual number of unconscious and infected patients hindered the relational dimension of care and increased the risk of burnout. A previous study showed that a high ICU morality rate was associated with a higher number of cases of burnout and depression [63]. In our study, this confrontation was a source of suffering for ICPs. Furthermore, the exceptionally high mortality rate in ICUs engendered in ICPs a feeling of helplessness, the feeling of performing useless and meaningless work, which increased the risk of brownout [27,33]. Once again, the decrease in the number of patients in the intensive care unit and, consequently, the decrease in workload, may explain why the risk factors for burnout related to care and the associated indicators were less present. It was assumed that the “care” dimension, which complements the “cure” dimension, once again played an important role in the care relationship at the end of the third wave [64]. The decrease in the number of deaths (less than 1500 cases between February and May 2021) is also likely to explain a decrease in the feeling of impotency [40,43]. Finally, like relations with patients, relations with other professionals in the service seemed to have deteriorated in the context of the health crisis. Indeed, our study results highlighted more inadequate behaviours, which weakened interpersonal relationships. This is a phenomenon of depersonalisation [17], which is particularly present when emotional exhaustion is too great [65]. Moreover, the lack of recognition leads to a deterioration in the quality of life at work [23,66]. The loss or the threat of the loss of recognition of one’s work can provoke anxiety, and these relational failures are a stress factor and constitute a burnout risk [57,67]. These psychosocial processes involve emotions, which in our study led ICPs to feel even more strongly and which were sometimes difficult to mask. This emotional work can engender burnout by suppressing the emotions actually felt [68]. 

Moreover, the relational dimension of care that constituted professional identity gradually slipped from the grasp of the ICPs participating in our study given the restrictions on family visits, which was considered unethical by our study population, or the unethical rules and tasks, contributing to the risk of brownout [30]. Providing real support, something made impossible by new health regulations and restrictions increased ICPs’ emotional burden and a feeling of guilt [69]. Under these conditions, virtual digital communication tools became essential in supporting families who had a family member in intensive care. More broadly, the crisis was an “accelerator of info-communication problems in the health sector” [70], p. 2. Despite being an opportunity for interaction and remote medical monitoring, their implementation raised concerns about the dehumanisation of patient care [70] and altered the proximity between health care providers and patients’ families, which can lead to unspoken words and a feeling of frustration [71]. The gradual reopening of the hospital’s doors to families between T1 and T2 made it possible to put ethical values and the human dimension back at the centre of patient care. The reduction of dissonance with personal values is likely to reduce the work-related brownout. However, re-establishing links with the families was not without its difficulties; the interactions were sometimes conflictual and violent, a factor of burnout already recognised in intensive care [23]. In addition, ethical conflicts related to decision-making about the management of COVID-19 patients persisted over time (T2). While ethical issues already represented risk factors for burnout and brownout prior to the COVID-19 health crisis, they were exacerbated in the times of the pandemic [30,72].

For the interns, the workload and the impact of the context on their training conditions led to uncertainty regarding their professional future and to a reconsideration of their perceived skills, in other words, to the occurrence of brownout [32]. While the difficulties linked to their lack of experience were already a factor for psychological disorder [73], the pandemic period may have led to a collapse in their professional prospects. However, with time, they realised that the confrontation with a new and challenging work context had also been an opportunity to acquire new skills and a certain expertise in this field.

Indeed, the impact of the COVID-19 pandemic was not only deleterious. Although few in number, some studies have reported the benefits the crisis brought to care providers’ work [74], in relation to the type and quality of care administered, support, patient recovery, and pride in their commitment. In our study, we noted a reinforced feeling of accomplishment in several ICPs; the lack of this feeling is one of the three components of burnout [17]. In addition, the ICPs reported that, at the beginning of the crisis, there was teamwork and a positive relationship between colleagues. Elsewhere, cohesion and team spirit were reported as essential elements to working together [75]. The recognition of work accomplished, the development of medical knowledge, and the reinforcement of professional skills are also two motivating factors intrinsically linked to work [76]. The feeling of having (re)found meaning to one’s work—something considered useful and even essential—through the provision of effective care was just as beneficial and prevented burnout [77].

Before concluding, it should be noted that one of the major limitations of this work was that, when we identified the two timeframes for our study, we did not know how the health crisis had evolved and were unaware of the relevance of the choice of these timeframes. We performed the first round of data collection during the third pandemic wave and, therefore, well after the beginnings of the crisis. We hypothesise that a longer period of time between two interviews and that conducting the first interviews (i.e., T1) as soon as the pandemic started would have made it possible to accentuate the longitudinal design or our study and to observe more salient developments.

## 5. Conclusions and Perspective

There is a common thread between the two types of professional exhaustion: burnout and brownout. The risk of both types occurring is heightened during health crises. The results of our study highlighted the need to pay particular attention to the health of ICPs, a population severely tested for two years, for whom the medium- and long-term repercussions of the COVID-19 are still difficult to assess.

Many care providers left their hospital positions during the COVID-19 crisis, either temporarily or permanently. Others still intend to. This brings us to wonder about future repercussions in terms of human resources, especially since the situation in hospitals and working conditions pre-COVID-19 were already a cause of concern. The ongoing crisis continues to give rise to legitimate concerns about new difficulties that hospitals will face.

Despite this, the positive effects of the crisis experience have redefined and even refocused the needs and priorities of ICPs. Their professional expectations translate into new objectives and a restructuring of professional identity to which responses will have to be provided to ensure, at least in part, well-being at work. In short, the deterioration of the health of ICPs, as well as their professional aspirations must be monitored carefully. This is why recommendations and measures should be designed and developed on how to support ICPs in “post-crisis” professional activity. Intervention research would seem a particularly appropriate approach to adopt.

Finally, our study highlights the relevance of questioning the different dimensions of mental health at work and the need to pay close attention to psychological disorders and tell-tale signs of possible problems, which result from the degradation of the subjective relationship to work.

## Figures and Tables

**Table 1:** Collective and individual indicators of burnout [19].

Collective Level		Individual Level
Indicators related to the operation of the structure	Indicators related to worker health and security	Lack of energy to perform the job, problems with concentration and lack of “mental” availability at work, irritability, denigration about the job or the work environment, devaluation of the work performed, one’s own effectiveness and skills, unusual signs of disinvestment and disengagement from work, emotional exhaustion
Working hours, staff turnover, absenteeism rate, company or structure activity, social relations	Activity of the occupational health service, occupational accidents, occupational diseases, serious or deteriorated situations, diagnosed and treated pathologies	

**Table 2:** The four dimensions of burnout in intensive care professionals [23].

Organisational Dimension	Relational Dimension	Care-Related Dimension	Workplace-Related Dimension
Working hours, overwork, understaffing, inexperienced staff, emergencies, admissions, transfers	Conflict in the team, disagreement on the management of patients, difficulties of cooperation between the different departments, lack of recognition	Severity of pathologies, lack of information, management of uncertainty, difficulty communicating with patients, conflict with patients’ families, decision-making, risk of error, perception of inadequate care	Noise, unsuitable premises, equipment problems

**Table 3:** Main indicators of brownout [30,31,32,33,34].

Authors	Main Indicators
Rigby, 2015	Mental resignation; progressive disinvestment and disengagement at work; feelings of disillusionment and despondency; feelings of lassitude; uncertainty about one’s professional future; lack of perspective on one’s professional career; psychological and emotional health (e.g., sleep disorders, irritability, etc.); disinvestment in family and social life; absenteeism
Alvesson and Spicer, 2016	Feeling disillusioned and downcast; gradual disinvestment; disengagement; resignation
Chapelle, 2018	Decrease in psychic tension; disinvestment; loss of commitment; dissatisfaction
Petiau, 2018	Work-related malaise
Knani and Gril, 2022	Mental resignation; disengagement; feeling disillusioned and down

**Table 4:** T1 (February 2022) and T2 (May 2022) interview guide.

Component Questions of the Interview Guide
How do you currently feel about your work? How would you describe your current state of health? What do you think is causing you to feel this way? On an emotional level, how do you deal with the context in which you work? Currently, how would you describe the working climate in your service? How would you describe the relationship you have with patients and their families? Can you tell me about decision making in the ICU? How do these decisions and the way they are made affect you and your work? Can you tell me about the way in which you articulate your private and professional life? At the moment, how do you see your career continuing?

**Table 5:** Characteristics of participants (T1) (N = 17).

Category		Frequency
Sex	Female	5
	Male	12
Status	Residents	5
	Qualified anaesthesiologists/intensivists	12
Age (years)	25–35	12
	35–45	4
	65–75	1
Time working in ICU (years)	0–5	7
	5–10	7
	10–15	2
	40–45	1
Professional practice	Anaesthesia and intensive care	3
	Intensive care only	14

**Table 6:** Characteristics of participants (T2) (N = 9).

Category		Frequency
Sex	Female	3
	Male	6
Status	Residents	3
	Qualified anaesthesiologists/intensivists	6
Age (years)	25–35	7
	35–45	2
Time working in ICU (years)	0–5	7
	5–10	1
	10–15	1
Professional practice	Anaesthesia and intensive care	1
	Intensive care only	8

**Table 7:** Articulation of categories and sub-categories based on a process of grounded theorisation.

Categories	Sub-Categories
The socio-health context and organisation of work during the COVID-19 pandemic	Work uncertainty and adaptation to changes Perceived characteristics of the work environment Social climate and well-being at work Work–life balance
The relationship with intensive care patients and their relatives, a new approach in the era of COVID-19	Use of ICT with patients’ families Quality of the care provider–patient relationship Ethical issues of care and access to care in the pandemic context
Multiple disorders of mental health: between burnout and brownout	Burnout Brownout
Resources contributing to maintaining good mental health	-

**Table 8:** Nature of burnout and brownout factors and indicators for T1 and T2 during the 3rd wave of the health crisis (T1 and T2).

Burnout Factors	Burnout Indicators	Brownout Factors	Brownout Indicators
Work uncertainty and adaptation to changes *	Individual level (emotional exhaustion, lack of energy to perform the job) *	Ethical issues of a new form of communication with patients’ families (ICT) *	Mental health (sleep disorders) *
Limits and ethical issues of a new form of communication with patients’ families (ICT) *	Individual level (depreciation of the work accomplished) *	Negative work environment and negative social climate ***	Uncertainty of professional career prospects ***
Increased workload and role conflicts ***	Individual level (irritability) **	Work uncertainty and incomprehension of the tasks to be accomplished ***	Feeling of uselessness and weariness, demotivation ***
Negative work environment, lack of consideration and recognition ***	Collective level (related to the functioning of the structure) ***	Ethical issues of care and access to care in the pandemic context ***	Dissatisfaction ***
Work–life conflict ***	Individual level (emotional exhaustion) ***		Feeling disillusioned and down ***
Questioning the meaning of the doctor–patient relationship: human and communicative aspects ***	Individual level (devaluation of one’s skills) ***		
Ethical issues of care and access to care in the pandemic context ***			

* Factors and/or indicators identified only in T1, ** factors and/or indicators identified only in T2, *** factors and/or indicators identified in T1 and T2.

**Table 10:** Evolution for T2 of burnout and brownout indicators and factors in the nine participants who provided data for both data collection periods (T1 and T2).

Type of Exhaustion	Burnout (BO)		Brownout (BWO)	
Interview 1	Indic. BO **^1^**	Fact. BO **^2^**	Indic. BWO **^2^**	Fact. BWO **^2^**
Interview 3	Indic. BO **^1^**	Fact. BO **^1^**	Indic. BWO **^3^**	Fact. BWO **^1^**
Interview 4	*	Fact. BO **^2^**	*	**
Interview 5	Indic. BO **^1^**	Fact. BO **^1^**	Indic. BWO **^1^**	Fact. BWO **^1^**
Interview 8	*	Fact. BO **^1^**	Indic. BWO **^1^**	Fact. BWO **^1^**
Interview 10	Indic. BO **^1^**	Fact. BO **^1^**	Indic. BWO **^1^**	Fact. BWO **^1^**
Interview 12	Indic. BO **^2^**	Fact. BO **^1^**	Indic. BWO **^1^**	Fact. BWO **^1^**
Interview 15	Indic. BO **^2^**	Fact. BO **^1^**	*	Fact. BWO **^2^**
Interview 17	*	**	Indic. BWO **^2^**	Fact. BWO **^1^**
No. of ICPs concerned	6	8	6	8

**^1^** presence of Fact. and/or Indic. for T1 and for T2, **^2^** presence of Fact. and/or Indic. for T1 only, **^3^** presence of Fact. and/or Indic. for T2 only, * no indicator, ** no factor.

## Data Availability

The datasets generated and/or analysed during the current study are not publicly available because the privacy of the individuals could be compromised, but are available from the corresponding author upon reasonable request.
